# Testing the efficacy of a couple-focused, tailored eHealth intervention for symptom self-management among men with prostate cancer and their partners: the study protocol

**DOI:** 10.1186/s13063-021-05948-5

**Published:** 2022-01-04

**Authors:** Lixin Song, Matthew E. Nielsen, Ronald C. Chen, Christine Rini, Thomas C. Keyserling, Eno Idiagbonya, Gail P. Fuller, Laurel Northouse, Mary H. Palmer, Xianming Tan

**Affiliations:** 1 School of Nursing, University of North Carolina (UNC), Chapel Hill, NC USA; 2 Lineberger Comprehensive Cancer Center, UNC, Chapel Hill, NC USA; 3 School of Medicine, UNC, Chapel Hill, NC USA; 4 University of Kansas Medical Center, Kansas, USA; 5 Northwestern University, Cancer Survivorship Institute and Department of Medical Social Sciences, Chicago, USA; 6 School of Nursing, University of Michigan, Ann Arbor, MI USA; 7 Gillings School of Global Public Health, UNC, Chapel Hill, NC USA

**Keywords:** Prostate cancer, Caregiver, eHealth, Symptom management, Quality of life, Randomized clinical trial, Social support, Stress, Coping, Health behavior

## Abstract

**Background:**

Men with localized prostate cancer often experience urinary, sexual, bowel, and hormonal symptoms; general distress; pain; fatigue; and sleep disturbance. For men in an intimate relationship, these symptoms disrupt couples’ relationships and intimacy. The symptoms also reduce quality of life for both men and their partners, who are often their primary caregivers. Management of the negative effects of cancer and its treatment is a significantly under-addressed supportive care need for these men and their intimate partners. To address these unmet supportive care needs, our interdisciplinary team developed and pilot tested the usability and feasibility of an evidence-based, couple-focused, tailored eHealth intervention, “Prostate Cancer Education & Resources for Couples” (PERC). Based on the adapted stress and coping theoretical framework and developed with stakeholder involvement, PERC aims to improve quality of life for both men and their partners by enhancing their positive appraisals, self-efficacy, social support, and healthy behaviors for symptom management.

**Methods:**

We will test the efficacy of PERC using a population-based, geographically and demographically diverse cohort in a randomized controlled trial. *Primary aim*: Assess if patients and partners receiving PERC will report greater improvement in their cancer-related quality of life scores than those in the control group (usual care plus the National Cancer Institute prostate cancer website) at 4, 8, and 12 months post-baseline. *Secondary aim*: Test if patients and partners in PERC will report significantly more positive appraisals and higher levels of coping resources at follow-ups than those in the control group. *Exploratory aim*: Determine if patient race and ethnicity, education, type of treatment, or couples’ relationship quality moderate the effects of PERC on patient and partner QOL at follow-ups.

**Discussion:**

This study will provide a novel model for self-managing chronic illness symptoms that impact couples’ relationships, intimacy, and quality of life. It addresses the National Institute of Nursing Research’s goal to develop and test new strategies for symptom self-management to help patients and caregivers better manage their illness and improve quality of life. It also responds to calls for programs from the Institute of Medicine and American Cancer Society to address treatment-related effects and improve survivors’ QOL.

**Trial registration:**

CT.gov NCT03489057

## Administrative information

Note: the numbers in curly brackets in this protocol refer to SPIRIT checklist item numbers. The order of the items has been modified to group similar items (see http://www.equator-network.org/reporting-guidelines/spirit-2013-statement-defining-standard-protocol-items-for-clinical-trials/).

**Table:** 

Title {1}	Testing The Efficacy of a Couple-focused, Tailored eHealth Intervention for Symptom Self-Management Among Men with Prostate Cancer and Their Partners: The Study Protocol
Trial registration {2a and 2b}.	CT.gov ID: NCT03489057.
Protocol version {3}	**May 28, 2020**.
Funding {4}	National Institute of Health National Institute of Nursing Research R01 NR016990, PI: Song.
Author details {5a}	[1] School of Nursing, University of North Carolina (UNC), Chapel Hill, NC; [2] Lineberger Comprehensive Cancer Center, UNC, Chapel Hill, NC; [3] School of Medicine, UNC, Chapel Hill, NC; [4] University of Kansas Medical Center; [5] Northwestern University, Cancer Survivorship Institute and Department of Medical Social Sciences, Chicago; [6] School of Nursing, University of Michigan, Ann Arbor, MI’ [7] Gillings School of Global Public Health, UNC, Chapel Hill, NC.
Name and contact information for the trial sponsor {5b}	University of North Carolina Lineberger Comprehensive Cancer Center, Mary Hayden O’Dwyer [mary_odwyer@med.unc.edu].
Role of sponsor {5c}	The sponsor played no part in study design; collection, management, analysis, and interpretation of data; writing of the report; and the decision to submit the report for publication.

## Introduction

### Background and rationale {6a}

Prostate cancer is the most frequently diagnosed non-skin cancer in men in the USA, with 191,930 new cases reported in 2020 [1]. Approximately 92% of diagnosed men have local or regional disease [2]. They are treated with curable intent (prostatectomy or radiation therapy, with or without hormonal therapy), but they often experience distressing treatment-related urinary, sexual, bowel, and hormonal symptoms (e.g., urinary incontinence and urinary urgency, erectile dysfunction, diarrhea, and hot flashes) that linger for months or years [3–7]. These symptoms also lead to emotional distress, fatigue [8], pain and sleep disturbance [5, 9], and reduced quality of life (QOL) [7, 10], all of which can be addressed by self-management. With an approximately 97.5% 5-year relative survival rate and median age of 66 at diagnosis [11], men with localized prostate cancer are likely to survive for many years and die from other illnesses [12–15]. [Note: Five-year relative survival rates describe the percentage of patients with a disease alive 5 years after the disease is diagnosed, divided by the percentage of the general population of corresponding sex and age alive after five years] Psychosocial behavioral self-management strategies (e.g., healthy diet and physical activities) are effective for reducing general symptoms [16, 17], decreasing overall and cancer-specific mortality [18–20], and improving QOL among aging populations (including cancer patients) [17, 21, 22].

For men who are in an intimate relationship, partners are a critical source of support. They are often the primary caregiver, and they play an important role in seeking and gathering information besides providing tangible care and emotional support [23]. Prostate cancer and treatment-related symptoms also reduce partners’ QOL [24, 25], leading to disruption in couples’ intimacy and relationships [26–29]. The adverse effect of symptoms on the QOL of partners may be as great or greater than on the QOL of the patient diagnosed with prostate cancer [24, 25]. Addressing the needs of both the men and their partners is therefore of particular concern because the QOL of both is significantly related [25, 30]; each one’s unmet needs impact their own and each other’s QOL [30, 31]. The national agenda of the American Cancer Society [32] and National Comprehensive Cancer Network [33, 34] have included calls for programs to manage the physical and psychosocial effects of prostate cancer and its treatment; promote healthy behaviors for survivors and their families; and ultimately improve their QOL.

To address the unmet needs for men and their intimate partners, our interdisciplinary team has developed a tailored, couple-focused eHealth intervention called *Prostate Cancer Education & Resources for Couples* (*PERC*). PERC was developed based on contributions from stakeholders (survivors, partners, and oncology care providers) [35], findings from efficacious interventions with prostate cancer survivors and partners ^16,17^, and empirical evidence [32, 34, 36–38]. Guided by an adapted stress and coping theoretical framework [39, 40], PERC aims to improve QOL for both men undergoing treatment and their partners by enhancing positive appraisals of illness and boosting self-efficacy, social support from multiple sources, and healthy behaviors for symptom management. PERC uses eHealth technologies to dramatically increase couples’ ability to access post-treatment supportive care whenever and wherever they feel comfortable accessing it. We tested the usability and feasibility of PERC and refined PERC in two pilot studies (UNC Cancer Prevention and Control Intervention Research Pilot Grant Award and UNC LCCC Population Sciences Developmental Research Award, PI: Song for both awards) [35].

### Objectives {7}

We are currently conducting a randomized clinical trial (RCT) to test the efficacy of PERC (R01 NR016990, PI: Song; CT.gov ID: NCT03489057, IRB: 17-0482). We plan to achieve the following specific aims.

#### Primary aim

The primary aim is to assess the efficacy of PERC for improving QOL among men undergoing treatment and their intimate partners. We hypothesize that men and their partners receiving PERC will report greater improvement in their cancer-related QOL scores than those in the control group (usual care plus the National Cancer Institute (NCI) prostate cancer website) at 4, 8, and 12 months post-baseline.

#### Secondary aim

The secondary aim is to test the effects of PERC on symptom appraisals and coping resources. We hypothesize that men and their partners receiving PERC will report greater improvement in secondary outcomes and positive appraisals of illness and coping resources (i.e., self-efficacy in symptom management, greater social support, and use of more healthy behaviors) at follow-ups than those in the control group.

#### Exploratory aim

The exploratory aim is to examine if men undergoing treatments’ race/ethnicity, education, type of cancer treatment, or couples’ relationship quality at baseline moderate the effects of PERC on patient and partner cancer-related QOL at follow-ups.

### Trial design {8}

**Fig. 1:**
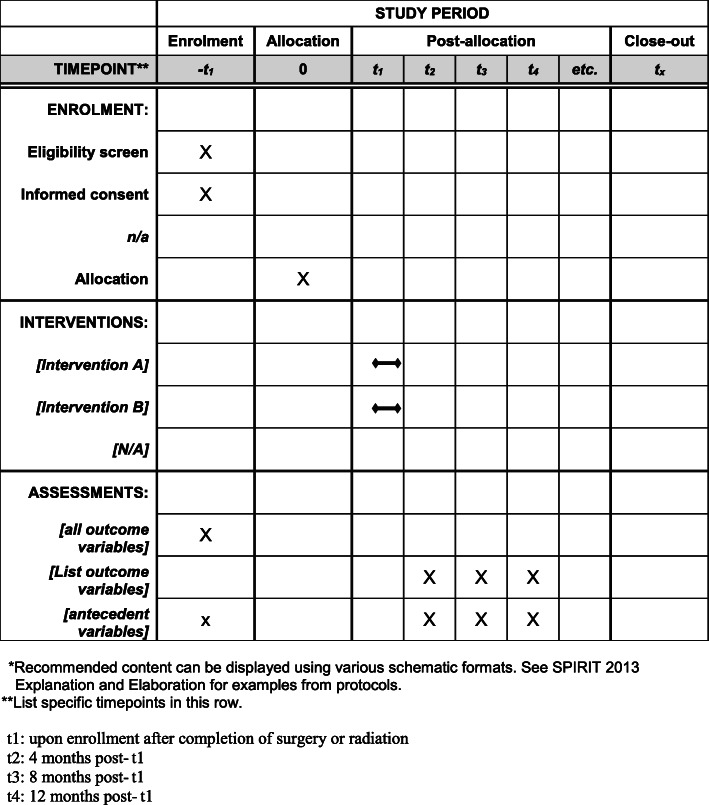
Example template of recommended content for the schedule of enrolment, interventions, and assessments

This is a population-based, statewide, two-arm, parallel groups RCT to test the efficacy of PERC to improve the QOL of men with prostate cancer who are post-treatment and their intimate partners. The eligible dyads are randomly assigned to PERC or to usual care plus the NCI website groups (Fig. 1).

## Methods: participants, interventions, and outcomes

### Study setting {9}

We recruit patient-partner couples using the North Carolina Central Cancer Registry Rapid Case Ascertainment (RCA). The RCA uses an accelerated process to capture new cases within a week of diagnosis. We conduct data collection surveys via telephone. During COVID-19, we added online surveys in response to participants’ need for flexible scheduling for data collection. The intervention is conducted online or via telephone, according to participants’ preference.

### Eligibility criteria {10}

Men are eligible if they (1) are 40–75 years of age and within 16 weeks of completing initial curative-intent treatment for localized prostate cancer (as confirmed by medical record), (2) have no prior cancer within the past 2 years, (3) are not currently receiving treatment for a concurrent cancer (excluding non-melanomatous skin cancer), (4) experience prostate cancer-specific and/or general symptoms (as assessed by screening questions), and (5) have an intimate partner (male or female) who is willing to participate. Intimate partners must be 18 years or older and not have been diagnosed with cancer or received treatment for cancer within the past 12 months; this criterion ensures that couples can focus their efforts on managing prostate cancer. Patients and partners must be able to read and speak English and they cannot have severe cognitive impairment. We will exclude men who are waiting for their initial treatment or on active surveillance.

### Who will take informed consent? {26a}

Trained research assistants who are responsible for participant enrollment and survey completion.

### Additional consent provisions for collection and use of participant data and biological specimens {26b}

We mail potential participants an introductory letter, a brochure, an opt-out letter (which they can mail back if not interested), and informed consent information. Our trained research assistants then call within 2 weeks of mailing the materials to assess interest in participating, answer questions, and screen for eligibility. We use the same procedure to screen partners’ eligibility after eligible men give permission for us to contact their partner. The research assistants obtain informed consent from men and their partners separately via telephone. All consents are audio recorded and saved in a password-protected encrypted folder. Participants can also choose to mail their signed informed consent forms back to the study team.

## Interventions

### Explanation for the choice of comparators {6b}

We use usual care plus the NCI prostate cancer website as the control condition. In usual care, patients access a range of sources (e.g., health providers, handouts and books, and online) to satisfy their information needs [41]. To ensure that our usual care participants access evidence-based, guideline-adherent information, we also give them access to the NCI website (http://www.cancer.gov/cancertopics/types/prostate) through the study website. The NCI website provides information about prostate cancer treatment options, research, causes, and statistics; coping resources that are not prostate cancer-specific; and support from non-providers via a toll-free phone and LiveHelp Online Chat about cancer-related questions, clinical trials, and quitting smoking. After logging in to the study website, usual care participants meet with the study educator at orientation using their preferred method (telephone or online) and then meet with the educator every 4 weeks via email. They receive general information about treatment and referral to the NCI website. Couples who fail to access the NCI website, as indicated by the Web activity tracking data, receive automatic messages via their preferred communication method (email, text, phone, or mail) to remind them to visit the website.

### Intervention description {11a}

In response to the increased use of technology to access information (including by older adults) [42–45] and to promote and help the uptake of PERC, we use eHealth technologies to improve access to the knowledge and skills that are critical and sensitive during care transitions for both patients and their partners. The PERC website includes three major sections: (1) 10 education modules with post-session assignments, (2) a moderated online Forum, and (3) a Resource Toolbox. We provide links to scientific publications in the Resource Toolbox to satisfy the learning needs of participants with high education levels. PERC uses clear, easy-to-read text; minimizes webpage navigation redirects; and incorporates audio-enhanced slides, videos, and graphics in each module to reach people at different reading and education levels and with different learning styles. The research staff provides ongoing technical support via telephone and online during 8 am to 9 pm Monday to Saturday. In-person visits can be arranged if needed.

PERC also facilitates informational, appraisal, and emotional support from partners, peers, and professionals during care transitions. PERC assists couple support between the patient and his partner through couple-focused modules and post-session assignments in which couples practice effective communication skills; share with each other personal perceptions of and experiences with different symptoms; and collaboratively develop strategies to minimize negative perceptions and effects of these symptoms on their lives. As strongly recommended by our pilot participants [35], couples are encouraged to log in to PERC from the same computer so that they can work together, although they can use the program separately at their convenience.

Participants can obtain peer and professional support using the online Forum moderated by our trained nurse educator who has extensive knowledge about prostate cancer and experiences working with men with prostate cancer and their families. Participants can also receive professional support by scheduling monthly virtual meetings (and additional meetings as needed) with the educator, either online or via a computer-assisted telephone call. At the meetings, which last 15–30 min, the educator helps couples review their symptoms and management, identifies goals to achieve, reviews the online modules if needed, troubleshoots any technical problems, and encourages couples to use the different components of the PERC program. These meetings consist of PERC orientation (week 1), completion of modules (weeks 2–14), and the final session (week 15).

### Criteria for discontinuing or modifying allocated interventions {11b}

Patients and their partners will become ineligible for further participation in this study if either or both receives a diagnosis of any type of cancer (e.g., breast, bowel) except for non-melanomatous skin cancer or develops a condition that prevents them from fully participating in study activities such as scheduling and completing surveys or online or computer-assisted telephone call with the nurse educator. Participants will also be removed from the study if they decide to withdraw from the study voluntarily.

### Strategies to improve adherence to interventions {11c}

We follow fidelity guidelines for health behavior research recommended by the NIH Behaviour Change Consortium [46] and recommendations to ensure fidelity for mHealth interventions [47].

#### Training

Before initiating PERC, we trained the nurse educator about the program, the PERC website, and process monitoring, using a standardized protocol checklist developed and refined in our pilot studies.

#### PERC implementation

Use of mHealth technology ensures that PERC is presented consistently. The nurse educator documents their intervention activities in the administrative log and records their online meetings with participants. The PI and project coordinator meets with the nurse educator to review fidelity issues and to provide feedback and demonstrations; these meetings occur weekly or whenever any member of the research team notices a discrepancy between the educator’s activity and the protocol.

#### PERC receipt

Our built-in Web activity tracking system assesses and records participant use of PERC. Participants who fail to enter the website are reminded via email, phone, or text messages. PERC was designed to minimize complexity by using plain language, using a low reading level, and implementing simple website navigation.

#### PERC enactment

The research team provides technical support to help address and document any issues that prevent participants’ use of PERC website.

### Relevant concomitant care permitted or prohibited during the trial {11d}

Participants are informed that this supportive care intervention does not interfere with their clinical care for cancer and/or other comorbid conditions. We anticipate that this is a minimal risk study. The PERC and NCI websites provide state-of-science resources that participants can use at their convenience. We will also refer them to their treating clinicians should any serious adverse medical or psychological event happen. No post-trial care is needed.

### Provisions for post-trial care {30}

There is no anticipated harm and compensation for trial participation. However, we plan to refine the intervention and/or develop new programs to address any care needs reflected through participants’ comments and feedback during the trial.

### Outcomes {12}

Primary outcomes include QOL total and subdomain scores*.* We use the 27-item Functional Assessment of Chronic Illness Therapy General Scale (FACT-G) to measure cancer-related QOL [48] and the physical, social, emotional, and functional domains of QOL [49]. Partners report their own cancer-related QOL using the partner’s version of FACT-G with modified wording [50].

Secondary outcomes include appraisal and coping resources*. Appraisals of illness—*patients’ and partners’ perceptions of the degree of threat associated with prostate cancer and related symptoms—are measured using a 20-item version of the Appraisal Scales, abstracted from the 32-item patient version and the 27-item caregiver versions of the measure [51]. Responses are provided on a 5-point Likert response scale [51, 52].

*Self-efficacy* is assessed by a 9-item modified version of the Lewis Cancer Self-Efficacy Scale, which measures confidence in managing prostate cancer symptoms. Responses are provided on a 10-point Likert scale. The partner version of the scale [53] evaluates partners’ confidence in their own ability to manage prostate cancer symptoms.

*Social support* is assessed using the PROMIS Emotional, Informational, and Instrumental Support Measures [54–56] and the appraisal support subscale of the Social Support Questionnaire [57].

*For healthy behaviors*, overall diet quality is assessed using the Dietary Risk Assessment (DRA) which is a brief food frequency dietary assessment [58]. Physical activity is assessed using the Measure of Older Adults’ Sedentary Time (MOST) [59, 60] which includes time spent in sedentary behaviors such as watching TV, computer use, reading, transport and hobbies, and total sedentary time.

Symptom outcomes. *Prostate cancer-specific symptoms* are measured using the Prostate Cancer Index Composite (EPIC-26) [61]. For patients, we will calculate [61] EPIC’s urinary irritability, urinary incontinence, bowel, sexual, and hormonal subscale scores. The partners complete a 4-item EPIC (spousal version), which assesses how much of a problem the patients’ bowel, hormonal, sexual, or urinary symptoms were for the partner.

*General symptoms*. Patients and partners separately rate their own symptoms of pain, fatigue, sleep disturbance [62–65], cancer anxiety [66], and depression [67] using the PROMIS measures.

### Participant timeline {13}

After informed consent and baseline assessments (T1, which occurs after completion of surgery or radiation), enrolled patient-partner couples are randomly assigned to PERC or enhanced usual care (usual care plus the NCI website) groups. After an intervention period that lasts approximately 15 weeks, participants complete three surveys to assess the short, intermediate, and long-term effects of PERC: at 4 months post-T1 (T2), 8 months post-T1 (T3), and 12 months post-T1 (T4).

### Sample size {14}

We planned to enroll 250 patient-partner couples to achieve the study objectives. We calculated power for comparing our primary outcome (total QOL) using a standard approach for linear mixed models [68]. Because we will assess outcomes for patients and partners separately, we applied a Bonferroni-corrected, two-sided alpha of 0.025 to allow for separate overall tests for patients and partners [68]. Although dyadic data will be modeled simultaneously, this correction allows for the possibility that conclusions may differ for patients and partners. Based on our pilot test of PERC [35], we assumed a common standard deviation for the total QOL scores of 15 points and a within-person correlation between repeated measurements of 0.75. Also, we allowed for losing up to 7% of participants every 4 months, for a total attrition of 20% through 12 months.

### Recruitment {15}

After receiving weekly reports of localized prostate cancer patients from North Carolina Central Cancer Registry Rapid Case Ascertainment (RCA), we contact patients’ physicians by letter and give them 2 weeks to request that a patient not be approached for study inclusion. After the 2-week window, we start to recruit using the procedure outlined above in the informed consent section.

## Assignment of interventions: allocation

### Sequence generation {16a}

After consented patients and partners independently complete the baseline survey, couples are randomized to PERC or the control group (usual care + NCI website) using a 1:1 ratio and stratified by type of treatment (surgery, radiation with or without hormonal therapy). Within each stratum, we use permuted block randomization with variable block size to generate allocation plan.

### Concealment mechanism {16b}

Our study biostatistician prepares and centrally maintains the computer-generated random numbers and uploads the randomization plan to the REDCap system.

### Implementation {16c}

Upon receiving the notice of a participant’s completion of baseline surveys, the interventionist (i.e., a nurse educator) obtains from REDCap the group allocation for the enrolled couples and informs them of their assignment to initiate intervention activities. After randomization, participants are guided to access their assigned website (PERC or NCI) via the study website using their assigned username and temporary password.

## Assignment of interventions: blinding

### Who will be blinded {17a}

Our research team members (i.e., the principal investigator, the co-investigators except the biostatistician, and the data collectors) are blinded to participants’ treatment allocation until the end of the study. The interventionist—the nurse educator—is not blinded but will not conduct surveys or interviews. The study participants are not blinded after they start to use their assigned eHealth program. We have no plan to unblind allocation to other team members before the end of the study. We have separate staff meetings for data collectors and the interventionist to discuss any participant-related issues and only use participants’ study IDs in our discussion to maintain blinding during the study period.

### Procedure for unblinding if needed {17b}

We have no plan to unblind allocation to other team members before the end of the study. We have separate staff meetings for data collectors and the interventionist to discuss any participant-related issues and only use participants’ study IDs in our discussion to maintain blinding during the study period.

## Data collection and management

### Plans for assessment and collection of outcomes {18a}

Research staff (data collectors) collect study data using a telephone survey at baseline (upon enrollment, T1) and at 4, 8, and 12 months post-T1. The data collectors are blinded to participants’ group assignment. During COVID-19, we added online surveys in response to participants’ needs for flexible scheduling for data collection.

### Plans to promote participant retention and complete follow-up {18b}

Since retention in longitudinal studies can be a challenge, we use Northouse and colleagues’ retention strategies [69] which were successful in our preliminary studies to maximize participant retention for both the PERC and control groups. We mail participants $20 per person after they complete each survey, with additional incremental compensation to help motivate them for longer-term participation ($10, $10, and $30 at the completion of the 4-, 8-, and 12-month post-baseline follow-ups, respectively). We also send participants retention gifts at 6 and 10 months. We also have regular team meetings to promptly address any issues that may negatively impact participant retention.

### Data management {19}

Telephone surveys are scripted and audio recorded, and data are entered simultaneously into the REDCap system, a secure, HIPAA-compliant database for data entry and management. The research team randomly checks at least 15–25% of the audio recordings against completed data for adherence to protocol, data completeness, and accuracy.

### Confidentiality {27}

To protect the confidentiality of participant data, this study minimizes uses of hardcopy research records; the PI and the independent Safety Officer ensure all hardcopy records are saved in a locked cabinet in a locked private office. With most data and documents being electronic, the PI and the Safety Officer ensure that the identifiable and de-identified data and documents are saved separately in different project folders in the password-protected and encrypted, shared drive at the university, which is on a secure university server. Only authorized key study personnel will have access to the identifiable information.

The electronic data include survey recordings and recordings of meetings between the nurse educator and study participants for quality control, as well as the de-identified survey data, study progress data and documents, and Web activity tracking data. The PI and the Safety Officer ensure that these data are tracked using study ID with no identifiable information attached. As a part of the university network and complying with university security regulations, we work closely with IT staff to ensure both security and efficiency for the study. Adverse event reports and annual summaries will not include information that can be used to identify participants. Each will include study IDs only.

### Plans for collection, laboratory evaluation, and storage of biological specimens for genetic or molecular analysis in this trial/future use {33}

**This trial does not involve collection, laboratory evaluation, or storage of biological specimens for genetic or molecular analysis.**

## Statistical methods

### Statistical methods for primary and secondary outcomes {20a}

Primary analyses will include all randomized participants, analyzed in the arm to which they are randomized, regardless of the extent of intervention received (intention-to-treat).

#### Primary aim

To assess the efficacy of PERC for improving QOL, we will compare the longitudinal mean change in overall QOL between groups using analysis of covariance (ANCOVA), conducted using linear mixed models. Data for patients and partners will be fit together in the same model (accounting for within-couple correlation). Each model will include fixed effects (separate for patient or partner) for group, month, group-by-month interactions, the baseline value of the outcome scale, baseline treatment type, number of baseline comorbidities, couple relationship quality, and demographics. Models will include random dyad and participant nested within dyad effects to account for within-dyad and within-person correlations between longitudinal responses. For the primary comparison, separately for each participant type, we will first test for any differences between groups across all 3 time points using an appropriately specified 3 degree of freedom linear contrast.

#### Secondary aim

To test the effects of PERC on appraisals and coping resources, we will use similar models to compare each of the QOL subdomains groups and to test the secondary outcome hypotheses. We will explore the potential mediating effects of appraisal and coping resources using a longitudinal path analysis model [70]. The model will include all appropriate within-dyad and longitudinal correlations.

### Interim analyses {21b}

We have no plan to conduct interim analyses because there are no anticipated problems that are detrimental to the participants.

### Methods for additional analyses (e.g., subgroup analyses) {20b}

#### Exploratory aim

The exploratory aim is to determine if patient race/ethnicity, education, type of cancer treatment, and couples’ relationship quality at baseline moderate the effects of PERC on patient and partner QOL at the follow-ups. We will test appropriate experimental group-by-moderator interactions using similar linear mixed models as specified for the primary aim. We will also analyze outcome and process data to identify critical characteristics of PERC participants, e.g., differences in race/ethnicity and education, in their PERC use patterns and outcomes.

### Methods in analysis to handle protocol non-adherence and any statistical methods to handle missing data {20c}

We closely follow the regulatory documentation and reporting process that are strictly implemented by the NIH and UNC IRB for any non-adherence and deviations. We use the intent to treat methods to handle missing data.

### Plans to give access to the full protocol, participant-level data, and statistical code {31c}

- The members of our research team will have access to the trial dataset that is deidentified once the trial is completed. The final dataset will be available for researchers who are interested in the related topics after the research team has disseminated the main findings of the research aims. Permission from the PI is required for any publications and dissemination effort.

## Oversight and monitoring

### Composition of the coordinating center and trial steering committee {5d}

The multidisciplinary steering committee members include researchers with expertise in clinical care and research in the areas of prostate cancer treatment, family-based intervention, symptom management, psychology, gerontology, digital health, and health disparities. The committee meet once a month during the first 2 years of the trial and then quarterly. The coordinating core team that runs the trial day-to-day including the PI, a project coordinator, a research nurse, and various numbers of research assistants who are responsible for enrolment and retention, as well as a data scientist. This core team meet at least once a week or more often if needed during the trial.

### Composition of the data monitoring committee, its role, and reporting structure {21a}

This study is a population-based, statewide randomized controlled efficacy trial evaluating a behavioral intervention in which prostate cancer patients who are post-initial treatment with curative intent and their partners use a Web-based symptom self-management intervention at their homes to enhance their knowledge and skills, promote social support, facilitate health behaviors, and ultimately improve their QOL. A data monitoring committee board (DSMB) is not needed because this study is a minimal risk study. However, we have implemented a data and safety monitoring plan for monitoring the trial with an executive committee of study leaders (PI and Co-Is) and study team members who implement study procedures to ensure the safety of participants as well as the validity and integrity of the data.

### Adverse event reporting and harms {22}

An Adverse Event Monitoring Committee has also been formed to oversee the conduct of the study. Chaired by the PI, the committee is comprised of the co-investigators. The committee meets regularly (monthly during the first 2 years of the study and then quarterly) to review the activities of the study. The research staff ensures all entry criteria are met prior to the initiation of the protocol. They also ensure that all study procedures and reporting of adverse events and unanticipated events is performed according to the IRB-approved protocol. All intervention-related adverse events will be reported to the IRB within 3–7 days. The PI will submit necessary reports to the funding agency. The PI and the Adverse Event Monitoring Committee assess the level of risk from adverse events (AEs) as mild (no interference in usual activities), moderate (some interference in usual activities), or severe (usual activities were significantly interrupted). The PI and the Adverse Event Monitoring Committee will assess whether events are related to the study using the following categories: not related, unlikely, possible, probable, or definite. We also hire an Independent Monitor who is independent from the present study design or implementation for data and safety monitoring. The Adverse Event Monitoring Committee will report adverse events to the Independent Monitor quarterly and when necessary to minimize research-associated risks.

### Frequency and plans for auditing trial conduct {23}

The PI convenes weekly meetings with the research staff to review project progress, subject accrual, follow-up, and any anticipated or unanticipated AEs. The PI and project manager are responsible for monitoring study processes and ensuring that AEs and unanticipated events (UEs) are reported immediately to the Adverse Event Monitoring Committee, the Independent Safety Officer, and the IRB within 3–7 days of the incidents. Additionally, the PI will report to the funding agency at NIH within 24 hours of the severe AEs and actions, if any, taken by investigators or the IRB because of the event or its continuing review. The study submits a complete data and safety monitoring summary report to the IRB as part of the annual renewal approval process and to the NIH with the annual progress report.

### Plans for communicating important protocol amendments to relevant parties (e.g., trial participants, ethical committees) {25}

We plan to notify the sponsor and funder first and then the steering committee and the core team. A copy of the revised protocol will be sent to the PI to add to the Investigator Site File. Any deviations from the protocol are fully documented using a breach report form and reported to IRB and sponsor protocol office. We also plan to update the protocol in the clinical trial registry.

## Dissemination plans {31a}

Dissemination has been considered in the design of PERC such as ease of use by participants and staff (with the possibility of removing the moderated online Forum after the efficacy testing). We are well positioned to disseminate the self-directed, eHealth PERC program by working with prostate cancer advocacy and support groups and by incorporating PERC into clinical care for men with prostate cancer and their partners. We will also disseminate the study results at national and international conferences and in peer-reviewed interdisciplinary journals.

## Discussion

This study will provide a novel model for self-managing chronic illness symptoms that impact couples’ relationships, intimacy, and quality of life. It addresses the National Institute of Nursing Research’s goal to develop and test new strategies for symptom self-management to help patients and caregivers better manage their illness and improve quality of life. It also responds to calls for programs from the Institute of Medicine and American Cancer Society to address treatment-related effects and improve survivors’ QOL.

## Trial status

The recruitment for this RCT began on May 30, 2018, and was completed on April 30, 2021. We plan to complete the follow-up surveys by July 1, 2022. The research team was busy with competing demands of daily operation of this R01 and other ongoing research projects before COVID-19 occurred in early 2020, which has significantly delayed all of our research activities, including publications.

The study protocol date: May 28, 2020.

## Abbreviations

RCT: Randomized clinical trial
QOL: Quality of life
PERC: PROSTATE cancer resources for couples
NIH: National Institutes of Health
AE: Adverse event
UE: Unexpected event
IRB: Institutional Review Board
PI: Principal investigator
Co-I: Co-investigator
NCI: National Cancer Institute
